# Cardiac valvular abnormalities associated with use and cumulative exposure of cabergoline for hyperprolactinemia: the CATCH study

**DOI:** 10.1186/s12902-020-0507-8

**Published:** 2020-02-19

**Authors:** Amer Budayr, Thida C. Tan, Joan C. Lo, Jonathan G. Zaroff, Grace H. Tabada, Jingrong Yang, Alan S. Go

**Affiliations:** 1 Division of Endocrinology, Kaiser Permanente Oakland Medical Center, Oakland, CA USA; 2 Division of Research, Kaiser Permanente Northern California, 2000 Broadway, Oakland, CA 94612 USA; 3 Division of Cardiology, Kaiser Permanente San Francisco Medical Center, San Francisco, CA USA; 4 Departments of Epidemiology, Biostatistics and Medicine, University of California, San Francisco, San Francisco, CA USA; 5 Departments of Medicine, Health Research and Policy, Stanford University School of Medicine, Stanford, CA USA

**Keywords:** Cabergoline, bromocriptine, hyperprolactinemia, heart valve diseases, risk factor, dopamine agonist

## Abstract

**Background:**

Whether lower dose cabergoline therapy for hyperprolactinemia increases risk of valvular dysfunction remains controversial. We examined valvular abnormalities among asymptomatic adults with hyperprolactinemia treated with dopamine agonists.

**Methods:**

This cross-sectional study was conducted among adults receiving cabergoline or bromocriptine for > 12 months for hyperprolactinemia and had no cardiac-related symptoms. Cardiac valve morphology and function were assessed from transthoracic echocardiograms at the study visit (except for two participants) with evaluation performed blinded to type and duration of dopamine agonist received.

**Results:**

Among 174 participants (mean age 49 ± 13 years, 63% women) without known structural heart disease before starting therapy, 62 received only cabergoline, 63 received only bromocriptine, and 49 received both. Median cabergoline use was 2.8 years in cabergoline only users and 3.2 years for those exposed to both cabergoline and bromocriptine; median bromocriptine use was 5.5 years in bromocriptine only users and 1.1 years for those exposed to both cabergoline and bromocriptine. Compared with bromocriptine only users (17.5%), regurgitation of ≥1 valve was more common for cabergoline only (37.1%, *P* = 0.02) but not for combined exposure (26.5%, *P* = 0.26). Compared with bromocriptine only exposure (1.6%), regurgitation of ≥2 valves was more common for cabergoline only (11.3%, *P* = 0.03) and combined exposure (12.2%, *P* = 0.04). Cabergoline only users had higher age-sex-adjusted odds for ≥1 valve with grade 2+ regurgitation compared to bromocriptine only users (adjusted odds ratio [aOR] 3.2, 95% confidence interval [CI]:1.3–7.5, *P* = 0.008), but the association for combined exposure to cabergoline and bromocriptine was not significant (aOR 1.7, 95%CI:0.7–4.3, *P* = 0.26). Compared to bromocriptine only, age-sex-adjusted odds of ≥2 valves with grade 2+ regurgitation were higher for both cabergoline only (aOR 8.4, 95% CI:1.0–72.2, *P* = 0.05) and combined exposure (aOR 8.8, 95% CI:1.0–75.8, *P* = 0.05). Cumulative cabergoline exposure > 115 mg was associated with a higher age-sex adjusted odds of ≥2 valves with grade 2+ regurgitation (aOR 9.6, 95%CI:1.1–81.3, *P* = 0.04) compared to bromocriptine only.

**Conclusions:**

Among community-based adults treated for hyperprolactinemia, cabergoline use and greater cumulative cabergoline exposure were associated with a higher prevalence of primarily mild valvular regurgitation compared with bromocriptine. Research is needed to clarify which patients treated with dopamine agonists may benefit from echocardiographic screening and surveillance.

## Background

Most patients with persistent hyperprolactinemia have a prolactin-secreting pituitary prolactinoma and are generally treated with dopamine agonists (bromocriptine or cabergoline) which can effectively reduce prolactin secretion and size of the lactotroph adenoma, if present. Bromocriptine is usually preferred in women with infertility given its relative safety during early pregnancy, but cabergoline is better tolerated and potentially more effective than bromocriptine [1]. Cabergoline is frequently first-line therapy in men, as well as in women who are not considering pregnancy.

However, previous studies reported an increased incidence of cardiac abnormalities in adults with Parkinson’s disease treated with high-dose dopamine agonists, [2–5] including cabergoline. Studies suggest that dopamine agonists with serotonin receptor agonist activity were associated with adverse cardiac valvular effects (i.e., valvular and chordal thickening) [6]. In 2007, the dopamine agonist, pergolide, was also removed from the market for valvular abnormalities [7]. Whether the risk is similar in conditions where lower doses of cabergoline are prescribed is still not well understood, with conflicting results in published studies [5, 8–17]. A recent meta-analysis of 13 case-control studies comparing patients treated with cabergoline for at least 6 months for hyperprolactinemia versus controls matched for selected patient characteristics reported higher odds of any tricuspid regurgitation but no other significant differences in other valves [18]. However, no included study in the meta-analysis had more than 102 patients treated with cabergoline, several studies examined only shorter-term cabergoline exposure, there was a wide range of cumulative cabergoline dosage across studies, and not all studies included blinded, standardized evaluations of echocardiograms [18].

Within a representative, community-based sample, our objective was to examine the relation between longer-term cabergoline use and cumulative dosage of cabergoline vs. bromocriptine use with the prevalence of cardiac valvular abnormalities in patients with hyperprolactinemia.

## Methods

### Source population and study sample

The source population was based in Kaiser Permanente Northern California, a large integrated health care delivery system currently providing comprehensive care to > 4.4 million members in the San Francisco and greater Bay area. The Kaiser Permanente Northern California membership is highly representative of the local surrounding and statewide population [19, 20].

In the Cabergoline, Other Dopamine Agonists, and Cardiac Health in Hyperprolactinemia (CATCH) Study, we first identified health plan members with diagnosed hyperprolactinemia identified from clinical databases between January 1, 1996 through December 31, 2006, and the diagnosis was confirmed by chart review by a board-certified endocrinologist using standardized clinical and biomarker criteria (A.B., J.C.L.). Patients had received filled outpatient prescriptions for dopamine agonist therapy (cabergoline, bromocriptine) from health plan pharmacies for ≥12 months as of December 2006 that was confirmed by telephone interview of the participant. We excluded patients if they met any of the following criteria: documented structural heart disease (i.e., diagnosed rheumatic heart disease, mitral stenosis or regurgitation, aortic stenosis or regurgitation, tricuspid regurgitation, prosthetic heart valve, heart failure, other structural heart disease) that was documented before starting dopamine agonist therapy by self-report or corresponding diagnosis or procedure codes found in medical records; absence of a pharmacy benefit plan with their health plan coverage which could lead to less than comprehensive dopamine agonist exposure capture; or inability to provide informed consent.

Using standard methods, we measured resting blood pressure (Welch-Allyn Vital Signs Monitor Model 5300P), height (stadiometer) and weight (Tanita digital scale). Participants also completed a questionnaire about sociodemographic characteristics, lifestyle factors, prior appetite suppressant and migraine therapy use, other medication exposures, and risk factors for valvular abnormalities.

### Characterizing exposure to dopamine agonists

We characterized longitudinal exposure to and cumulative dose of cabergoline and bromocriptine before the study visit based on dispensed prescriptions found in health plan pharmacy databases. Prescription information on pill count and estimated day supply, along with refill intervals, were used to estimate timing and duration of drug exposure and confirmed by manual review of individual refill patterns by a board-certified endocrinologist (J.C.L.). Based on this information, participants were classified as having received only cabergoline, only bromocriptine, or exposure to both cabergoline and bromocriptine (i.e., combined use).

### Evaluation of valvular morphology and function

Screening transthoracic echocardiography included a subset of images obtained using an Acuson Cypress® sonograph. Two dimensional (2D) settings were optimized to produce the highest resolution images of the cardiac valves. From the parasternal window, 2D images of valves were obtained, along with color flow Doppler (CFD) regurgitation signals for all four valves and continuous wave Doppler (CWD) profiles for the tricuspid and pulmonic valves. From the apical window, 2D images of the aortic, mitral, and tricuspid valve were obtained using the four, five, two, and three-chamber views. CFD regurgitation signals and CWD profiles were obtained for the aortic, mitral, and tricuspid valves. Pulse wave Doppler (PWD) signals were obtained for mitral inflow and pulmonary venous flow. In the subcostal view, a 2D clip of the inferior vena cava was obtained during inspiratory effort along with PWD profiles of hepatic venous and abdominal aortic flow. Zoomed in views of each valve in each view were included. All spectral PWD and CWD recordings were obtained at a sweep speed of 100 mm/sec. Nyquist limits were adjusted to the range of 0.5–0.7 m/sec.

For all participants, the echocardiographic core lab performed off-line analysis of image files imported into a ProSolv® database by a single cardiologist blinded to all clinical data, including any information about the type, dosing or duration of dopamine agonist therapy. For each cardiac valve, leaflet thickening and degree of regurgitation were semi-quantitatively assessed based on American Society of Echocardiography recommendations [20, 21]. Valvular regurgitation was classified as absent (0), trace (1), mild (2), moderate (3) or severe (4). Leaflet thickness was defined as normal (≤5 mm throughout), mildly thickened (6–8 mm focally or diffusely), or moderately to severely thickened (> 8 mm focally or diffusely) [22, 23]. Any valve labeled as “not well seen/visualized” or “not adequately visualized” was considered inconclusive and counted as not having a valvular abnormality in our analyses.

Echocardiograms for all but two participants were performed at the time of the study visit. For one participant, the echocardiogram was obtained shortly after the study visit, and for the other participant who was unable to attend a study visit, a clinically obtained echocardiogram performed within 12 months before their study visit was included and reviewed by the study echocardiographer (J.G.Z.).

### Statistical approach

Analyses were conducted using SAS version 9.4 (Cary, NC) and a two-sided *P* < 0.05 was considered significant. We compared characteristics across groups using Wilcoxon rank sum or chi-squared tests. We next calculated the prevalence of echocardiographic valvular abnormalities with associated 95% confidence limits in each treatment exposure group. For valvular regurgitation, we separately assessed if ≥1 valve and ≥ 2 valves were affected. In cabergoline users, we also examined the prevalence of significant valvular abnormalities by median cumulative dose. We used logistic regression to examine the age-sex-adjusted association between type of dopamine agonist received and prevalent valvular abnormalities, with bromocriptine only use as the referent group. We next used logistic regression to evaluate the age-sex-adjusted association between cumulative dose of cabergoline and prevalent valvular abnormalities compared with receiving bromocriptine. We conducted sensitivity analyses to examine the association between type of dopamine agonist exposure and the presence of only mild valvular regurgitation. Finally, we conducted additional sensitivity analyses to examine the association between dopamine agonist type and presence of grade 2+ valvular regurgitation among patients with no concomitant valve thickening.

## Results

### Baseline characteristics

In 2007, we enrolled 174 eligible asymptomatic patients with mean age 48.9 years and 63% being women (Table 1). Thirty-six percent of participants had received only bromocriptine, 36% only cabergoline and the remaining 28% received both. Age distribution did not significantly vary across groups, while cabergoline only participants were less likely to be women and combined users were more likely to be black (Table 1). Among participants who received only cabergoline, median use ranged from 2.8 years; among those who received only bromocriptine, median use was 5.5 years; while among those who received both cabergoline and bromocriptine, median use was 3.2 years for cabergoline and 1.1 years bromocriptine. Very few participants reported prior appetite suppressant drug use and a history of cardiac disease or risk factors for valvular disease before initiating dopamine agonist therapy were uncommon and did not differ across groups (Table 1).

**Table 1:** Baseline characteristics of 174 adults receiving therapy for hyperprolactinemia

Characteristic	Overall (*n* = 174)	Type of Hyperprolactinemia Therapy		
		Bromocriptine (*N* = 63)	Cabergoline (*N* = 62)	Cabergoline and Bromocriptine (*N* = 49)
Age, years				
Median (IQR)	47.3 (39.8, 56.8)	48.2 (42.3, 59.3)	46.7 (39.4, 57.7)	48.2 (36.7, 54.6)
Mean ± SD	48.9 ± 12.6	50.3 ± 12.6	48.7 ± 13.1	47.2 ± 12.1
Age Group, n (%)				*p* = 0.0743
21–40 years	50 (28.7)	13 (20.6)	19 (30.7)	18 (36.7)
41–60 years	90 (51.7)	35 (55.6)	32 (51.6)	23 (46.9)
> 60 years	34 (19.5)	15 (23.8)	11 (17.7)	8 (16.3)
Women (%)	110 (63.2)	43 (68.3)	29 (46.8) *	38 (77.6)
Race, n (%)				‡
White / European	75 (43.1)	29 (46.0)	28 (45.2)	18 (36.7)
African American / Black	33 (19.0)	5 (7.9)	9 (14.5)	19 (38.8)
Hispanic / Latino	26 (14.9)	13 (20.6)	10 (16.1)	3 (6.1)
Asian	32 (18.4)	11 (17.5)	12 (19.4)	9 (18.4)
Native American	2 (1.2)	1 (1.6)	1 (1.6)	0
Other	6 (3.5)	4 (6.4)	2 (3.2)	0
Body-Mass Index, kg/m^2^, n (%)				
< 20	7 (4.1)	1 (3.2)	1 (1.6)	5 (8.2)
20–24.9	46 (26.4)	16 (25.4)	17 (27.4)	13 (26.5)
25–29.9	41 (23.6)	15 (23.8)	21 (33.9)	5 (10.2)
≥ 30	79 (45.4)	29 (46.0)	23 (37.1)	27 (55.1)
Unknown	1 (0.6)	1 (1.6)	0	0
Smoking History				
Currently smoking, n (%)	9 (5.2)	3 (4.8)	3 (4.8)	3 (6.1)
Cigarettes per day, median (IQR)^b^	18.0 (6.0, 20.0)	18.0 (2.5, 20.0)	20.0 (20.0, 20.0)	6.0 (1.0, 10.0)
Years of smoking, median (IQR)^c^	15.0 (6.0, 30.0)	6.0 (3.0, 15.0)	30.0 (20.0, 32.0)	15.0 (1.0, 43.0)
Previously smoked, n (%)	54 (31.0)	22 (34.9)	21 (33.9)	11 (22.5)
Medication History				
Filled prescriptions, n (%)^d^	160 (92.0)	60 (95.2)	54 (87.1)	46 (93.9)
Cabergoline, median (IQR)				
Weekly Dose prescribed (mg)	1.0 (0.5, 1.0)	n/a	0.75 (0.5, 1.0)	1.0 (0.5, 1.0) ^‡ a^
Estimated Cumulative Dose (mg)	115.3 (52.2, 259.1)	n/a	98.0 (56.7, 214.0)	147.6 (45.4, 321.0)
Length of time, in years	2.9 (1.6, 5.2)	n/a	2.8 (1.9, 4.8)	3.2 (1.4, 6.1)
No. of prescriptions filled (per person)	15.0 (8.0, 24.0)	n/a	15.0 (10.0, 20.0)	14.0 (6.0, 29.0) *^a^
Bromocriptine, median (IQR)				
Daily Dose prescribed (mg)	2.5 (2.5, 5.0)	2.5 (2.5, 5.0)	n/a	5.0 (2.5, 7.5) ^‡^
Estimated Cumulative Dose (mg)	3711.9 (1023.8, 10,026.7)	6065.0 (2413.5, 11,630.0)	n/a	1127.5 (392.5, 4305.0) ^‡^
Length of time, in years	2.9 (1.1, 7.2)	5.5 (2.4, 9.8)	n/a	1.1 (0.5, 2.5) ^‡^
No. of prescriptions filled (per person)	14.5 (5.0, 36.0)	26.0 (10.0, 44.0)	n/a	5.0 (2.0, 16.0) ^‡^
Current Medication Usage (self-reported)				
Cabergoline current user, n (%)	96 (55.2)	n/a	57 (91.9)	39 (79.6)
Length of time (years), median (IQR) ^e^	5.0 (2.0, 6.9)	n/a	4.3 (2.0, 6.4)	5.0 (2.1, 8.0)
No. of days per week, median (IQR) ^f^	2.0 (1.0, 2.0)	n/a	2.0 (1.0, 2.0)	2.0 (1.0, 2.0)
Days per week varies, n (%)^g^	20 (11.5)	n/a	8 (14.5)	11 (22.5)
Bromocriptine current user, n (%)	63 (36.2)	57 (90.5)	n/a	6 (12.2) ^‡^
Length of time (years), median (IQR)	9.0 (4.5, 15.0)	9.0 (4.5, 15.0)	n/a	9.0 (4.8, 10.0)
No. of days per week, median (IQR)	7.0 (7.0, 7.0)	7.0 (7.0, 7.0)	n/a	7.0 (7.0, 7.0)
No. of days per week varies, n (%)	7 (4.0)	5 (7.9)	n/a	2 (4.1)
Amount taken varies, n (%)^h^	12 (6.9)	11 (17.5)	n/a	1 (2.0)
**Pregnancy**	*n = 110 women*	*n = 43 women*	*n = 29 women*	*n = 38 women*
Stopped taking due to pregnancy, n (%)	23 (20.9)	10 (23.3)	5 (17.2)	8 (21.1)
No. of times pregnant, median (IQR)	2.0 (1.0, 2.0)	2.0 (1.0, 2.0)	1.0 (1.0, 2.0)	2.0 (1.0, 3.0)
Breastfeeding duration, mos, median (IQR)	2.0 (0, 4.0)	2.0 (0, 5.5)	3.0 (2.0, 3.0)	1.0 (0.0, 4.3)
Risk Factors				
Migraines				
Treated, n (%)	24 (13.8)	8 (12.7)	7 (11.3)	9 (18.4)
Type of drugs taken, n (%)				
Dihydroergotamine, DHE 45	0	0	0	0
Ergotomone, Ergomar	1 (0.6)	0	1 (1.6)	0
Unknown	7 (4.0)	2 (3.2)	3 (4.8)	2 (4.1)
Weight Loss / Appetite Suppressant Drugs				
Taken to Suppress Appetite, n (%)	8 (4.6)	2 (3.2)	2 (3.2)	4 (8.2)
Type of drugs taken, n (%)				
Phentermine	0	0	0	0
Dexfenfluramine	0	0	0	0
Other	8 (4.6)	2 (3.2)	2 (3.2)	4 (8.2)
Unknown / forgot	5 (62.5)	2 (100.0)	0	3 (75.0)
Nano Slim Haudia	1 (12.5)	0	1 (50.0)	0
GNC BNRN 60	1 (12.5)	0	0	1 (25.0)
Hydroxycut	1 (12.5)	0	1 (50.0)	0
None	166 (95.4)	61 (96.8)	58 (93.6)	47 (95.9)
Medical History, n (%)				
Endocarditis	0	0	0	0
Rheumatic Fever	1 (0.6)	1 (1.6)	0	0
Mitral Valve Prolapse	2 (1.2)	0	1 (1.6)	1 (2.0)
Heart Failure	1 (0.6)	1 (1.6)	0	0
Myocardial Infarction	1 (0.6)	1 (1.6)	0	0
High blood pressure	23 (13.2)	8 (12.9)	7 (11.1)	8 (16.3)

* *P* value < 0.05; ^†^ *P* value < 0.01; ^‡^ *P* value < 0.001

^a^ Compares cabergoline to both users

^b^ Mean number of cigarettes smoked per day

^c^ Total number of years of smoking at average number of cigarettes per day

^d^ Number of patients who filled all their prescriptions with Kaiser Permanente pharmacies

^e^ Total years patient has taken drug

^f^ Mean number of days per week patient takes medication

^g^ Number of patients varying the number of days per week medication is taken

^h^ Number of patients whose medication amount taken varies

### Echocardiographic abnormalities

Overall, 11% of participants had mild aortic valve thickening and 10% had mild mitral valve thickening (Table 2). No participants had worse than mild aortic, mitral or pulmonic valvular thickening, and no participants had valvular thickening of the tricuspid valve.

**Table 2:** Characterization of valvular function by transthoracic echocardiography among 174 adults receiving therapy for hyperprolactinemia

Valve location	Valvular Thickening N (%)				Valvular Regurgitation N (%)				
	None	Mild	>Mild	Inconclusive	0	1	2	3	4
Aortic	155 (89.1)	19 (10.9)	0	0	14 (8.1)	154 (88.5)	6 (3.5)	0	0
Mitral	157 (90.2)	17 (9.8)	0	0	3 (1.7)	164 (94.3)	6 (3.5)	1 (0.6)	0
Pulmonic	150 (86.2)	1 (0.6)	0	23 (13.2)	15 (8.6)	133 (76.4)	26 (14.9)	0	0
Tricuspid	174 (100.0)	0	0	0	12 (6.9)	136 (78.2)	26 (14.9)	0	0

Only 3.5% of participants had grade 2 aortic valvular regurgitation, 4.0% had grade 2+ mitral valve regurgitation, 14.9% had grade 2 pulmonic valve regurgitation, and 14.9% had grade 2 tricuspid valve regurgitation (Table 2). Overall, 27.0% of participants had ≥1 valve with grade 2+ valvular regurgitation, 8.1% had ≥2 valves with grade 2+ valvular regurgitation; three participants had grade 2+ regurgitation in ≥3 valves and one participant had grade 2+ regurgitation involving all four values. The distributions of individual valvular lesions by exposure group are shown in *Supplemental Tables* *S1-S3*.

There were no significant differences in the prevalence of mild or greater valvular thickening by dopamine agonist exposure group (data not shown). Compared with bromocriptine only users (17.5%), cabergoline only users were more likely to have ≥1 valve with grade 2+ regurgitation (37.1%, *P* = 0.02), while there was no statistically significant difference for combined users (26.5%, *P* = 0.26) compared with participants who only received bromocriptine. Compared with bromocriptine only users (0%), 6.5% of participants exposed to only cabergoline had grade 2+ aortic valve regurgitation (*P* = 0.06), while 4.1% of combined users had grade 2+ aortic valve regurgitation (*P* = 0.19). Compared with bromocriptine only users (4.8%), grade 2+ pulmonic valve regurgitation was found in 24.2% of cabergoline only users (*P* < 0.01) and 16.3% in combined users (*P* = 0.06). There were no significant differences in the prevalence of grade 2+ regurgitation in mitral or tricuspid valves by treatment group (data not shown). Cabergoline only (11.3%, *P* = 0.03) and combined users (12.2%, *P* = 0.04) were also more likely to have ≥2 valves of any type with grade 2+ regurgitation compared with bromocriptine only users (8.1%), but there were no significant differences for any specific combination of valves between groups (data not shown).

After adjustment for age and sex, cabergoline only use was associated with higher odds of ≥1 valve of any type with grade 2+ regurgitation compared with bromocriptine only users (adjusted odds ratio 3.2, 95% confidence interval [CI]:1.3–7.5, *P* = 0.008), but the excess risk associated with combined exposure was not statistically significant (adjusted odds ratio 1.7, 95% CI:0.7–4.3, *P* = 0.26). Compared with bromocriptine only users, cumulative cabergoline exposure ≤115 mg was associated with higher age-sex-adjusted odds of ≥1 valve of any type with grade 2+ regurgitation (adjusted odds ratio 2.7, 95% CI:1.2–6.5, *P* = 0.02). Cumulative cabergoline exposure > 115 mg was also associated with higher age-sex-adjusted odds of ≥1 valve of any type with grade 2+ valvular regurgitation compared with bromocriptine users (adjusted odds ratio 2.1, 95% CI:0.8–5.0, *P* = 0.11) but was not statistically significant.

When examining the presence of ≥2 valves with grade 2+ valvular regurgitation, cabergoline only users had higher age-sex-adjusted odds compared with bromocriptine only users (adjusted odds ratio 8.4, 95% CI: 1.0–72.2, *P* = 0.05), as was combined exposure (adjusted odds ratio 8.8, 95% CI: 1.0–75.8, *P* = 0.05) that was of borderline significance. Compared with bromocriptine only users, cumulative cabergoline exposure > 115 mg was associated with a higher age-sex adjusted odds of ≥2 valves with grade 2+ valvular regurgitation (adjusted odds ratio 9.6, 95% CI:1.1–81.3, *P* = 0.04), while the association with cumulative cabergoline exposure ≤115 mg was of similar magnitude but of borderline statistical significance (adjusted odds ratio 7.7, 95% CI: 0.9–66.6, *p* = 0.06).

Results in sensitivity analyses examining associations of dopamine agonists restricted to only grade 2 valvular regurgitation did not materially differ from the main analyses (*Supplemental Tables* *S4-S7*). Additionally, sensitivity analyses examining associations of dopamine agonist therapies with grade 2+ valvular regurgitation among patients who did not have valve thickening also did not materially differ from the main analyses (results not shown).

## Discussion

In a diverse, community-based sample of patients receiving dopamine agonist therapy for hyperprolactinemia and no pre-treatment history of structural heart disease or active cardiac-related symptoms, those exposed to cabergoline were more likely to have at least mild valvular regurgitation compared with bromocriptine on screening transthoracic echocardiography, even after adjustment for age and sex. Cumulative cabergoline exposure > 115 mg (equivalent to as little as 2 years of therapy with a frequently used regimen of 1 mg per week) was associated with a notably higher age-sex-adjusted odds of primarily mild valvular regurgitation involving multiple valves.

Our results support and extend prior studies examining dopamine agonist therapy and valvular dysfunction. In a case-control study of individuals age 40 to 80 years old receiving ≥2 prescriptions for an antiparkinsonian medication (31 cases, 633 controls), [3] cabergoline was associated with higher odds of any valvular regurgitation (odds ratio 4.9, 95% CI:1.5–15.6). In addition, the odds of valvular regurgitation with cabergoline was higher for 3 mg/d (odds ratio 50.3, 95% CI:6.6–381.4) vs. < 3 mg/d (odds ratio 2.6, 95% CI:0.5–12.8). However, echocardiographic evaluation was only performed in symptomatic patients in that study, and patients receiving more than one dopamine agonist agent were assigned according to the agent with longest treatment duration. In another study of patients treated at a Parkinson’s disease clinic, those receiving cabergoline were more likely to have valvular abnormalities (relative risks 4.6–7.3 depending on the valve) [2]. In another small study of Turkish patients with Parkinson’s disease (34 treated with cabergoline, 42 with no treatment), both cumulative cabergoline dose and duration were associated with a composite valvular regurgitation score [22]. The mechanism of this potential effect may relate to the affinity of several dopaminergic agonists (including cabergoline) to the 5HT-2B receptor [24]. Bromocriptine may have a much lower agonist activity for the serotonin receptor compared with other dopamine agonists (e.g., pergolide and cabergoline) [24]. Importantly, nearly all studies have not observed an association between high-dose bromocriptine and valvular heart disease in patients with Parkinson’s disease [25].

Unlike patients with Parkinson’s disease, those with hyperprolactinemia generally require much lower doses of cabergoline (up to 1–2 mg/week) and are generally much younger (mean age 20–30 years), although treatment is typically continued for many years. Thus, hyperprolactinemia patients would need three to four decades of treatment with cabergoline to achieve cumulative exposure at the level where the risk of valvular abnormalities increased in studies of pergolide in patients with Parkinson’s disease [2, 5]. Interestingly, however, we found that a median cumulative exposure of only > 115 mg cabergoline over a relatively modest exposure time (median 2.8–3.2 years) was associated with higher age-sex-adjusted odds of primarily mild valvular regurgitation involving two or more valves compared with receiving only bromocriptine. Other studies in patients with hyperprolactinemia have found either no association of dopamine agonists with valvular abnormalities [5, 8–12, 26, 27] or a higher prevalence of regurgitation, generally involving the tricuspid valve, [5, 13–18] with no relation to the dose, duration of treatment, or cumulative dose [9, 24, 28]. Other studies have reported an association between long-term cabergoline exposure and aortic valve calcification in this population [29, 30]. However, the majority of studies included modest numbers of patients, primarily included only mild valvular regurgitation, did not include a comparison with patients treated with bromocriptine or involved patients from selected practice settings or referral clinics and/or had limited sociodemographic diversity.

Our study was strengthened by comparing valvular abnormalities in a diverse sample of patients with hyperprolactinemia treated with different dopamine agonists derived from a large, community-based source population receiving comprehensive care in an integrated health care delivery system. Another strength was the use of a single echocardiography reader who was blinded to the type, dose and duration of dopamine agonist therapy exposure and who implemented standardized criteria for evaluating the presence and severity of valvular abnormalities. Another contribution of our study is our ability to ascertain the dispensed dose of dopamine agonist therapies by reviewing detailed pharmacy information with validation by manual review of electronic medical records, which facilitated a more accurate estimate of cumulative drug exposure. However, the relatively low prevalence of specific valvular abnormalities precluded investigation of the association of different dopamine agonists for specific valvular dysfunction. In addition, all but one of the cases of valvular regurgitation in this cross-sectional study of asymptomatic adults was mild and the cumulative cabergoline exposure was lower than in several previous studies, so the clinical significance of these findings is less clear in this population. Given our sample size and distribution of longitudinal exposure to cabergoline, we also had limited precision for examining the association of cumulative cabergoline dose and valvular abnormalities. In addition, an echocardiogram that was clinically obtained during the 12 months before the study visit was included for analysis in one participant. As an observational study of outcomes associated with the two dopamine agonists, we also cannot rule out residual confounding as an explanation for our findings. Finally, as we did not have echocardiographic information on patients with hyperprolactinemia who were not treated with dopamine agonist therapy, we are unable to compare the prevalence of echocardiographic abnormalities in untreated patients.

## Conclusions

We found that compared with bromocriptine, cabergoline use and higher cumulative cabergoline exposure were associated with a higher prevalence of having primarily mild valvular regurgitation. Given the known risks of valvulopathy associated with carcinoid syndrome [31] and pergolide, [2] the effects of cabergoline on the 5HT-2B receptor, as well as conflicting findings in the existing literature, even larger additional studies with longer-term exposure of observed daily doses are needed among more contemporary, ethnically diverse populations to confirm our findings, to address the risk associated with different thresholds of cumulative cabergoline exposure, and to examine their potential long-term clinical significance. Furthermore, validated risk prediction models may assist patients and providers to understand the absolute risks of valvular disease associated with different dopamine agonists and support more evidence-based monitoring strategies for identifying the subset of patients with hyperprolactinemia who may benefit from targeted screening and surveillance strategies.

## Supplementary information

**Additional file 1: Table S1.** Valvular characteristics by transthoracic echocardiography among 63 adults receiving bromocriptine only therapy for hyperprolactinemia. **Table S2.** Valvular characteristics by transthoracic echocardiography among 62 adults receiving cabergoline only therapy for hyperprolactinemia. **Table S3.** Valvular characteristics by transthoracic echocardiography among 49 adults who received both bromocriptine and cabergoline therapies for hyperprolactinemia. **Table S4.** Age-sex-adjusted association of ≥1 valve with grade 2 regurgitation among patients treated with cabergoline only or with both cabergoline and bromocriptine (vs. only bromocriptine). **Table S5.** Age-sex-adjusted association of ≥1 valve with grade 2 regurgitation and cumulative cabergoline dose among patients treated with cabergoline only or with both cabergoline and bromocriptine (vs. only bromocriptine). **Table S6.** Age-sex-adjusted association between ≥2 valves with grade 2 regurgitation among patients treated with cabergoline only or with both cabergoline and bromocriptine (vs. only bromocriptine). **Table S7.** Age-sex-adjusted association between ≥2 valves with grade 2 regurgitation and cumulative cabergoline dose among patients treated with cabergoline only or with both cabergoline and bromocriptine (vs. only bromocriptine).

## Data Availability

The datasets generated and/or analysed during the current study are not publicly available or by request to the corresponding author due to constraints of the written informed consent obtained from participants at the time the study was conducted.

## Abbreviations

2D: Two-dimensional
CFD: Color flow Doppler
CI: Confidence interval
CWD: Continuous wave Doppler
PWD: Pulse wave Doppler
