# Supplementary material for: Cardiac valvular abnormalities associated with use and cumulative exposure of cabergoline for hyperprolactinemia: the CATCH study
Source: BMC Endocr Disord. 2020 Feb 19;20:25. doi: 10.1186/s12902-020-0507-8 (PMC7031983; doi:10.1186/s12902-020-0507-8)
Supplement: Supplementary file 1 — Additional file 1: Table S1. Valvular characteristics by transthoracic echocardiography among 63 adults receiving bromocriptine only therapy for hyperprolactinemia. Table S2. Valvular characteristics by transthoracic echocardiography among 62 adults receiving cabergoline only therapy for hyperprolactinemia. Table S3. Valvular characteristics by transthoracic echocardiography among 49 adults who received both bromocriptine and cabergoline therapies for hyperprolactinemia. Table S4. Age-sex-adjusted association of ≥1 valve with grade 2 regurgitation among patients treated with cabergoline only or with both cabergoline and bromocriptine (vs. only bromocriptine). Table S5. Age-sex-adjusted association of ≥1 valve with grade 2 regurgitation and cumulative cabergoline dose among patients treated with cabergoline only or with both cabergoline and bromocriptine (vs. only bromocriptine). Table S6. Age-sex-adjusted association between ≥2 valves with grade 2 regurgitation among patients treated with cabergoline only or with both cabergoline and bromocriptine (vs. only bromocriptine). Table S7. Age-sex-adjusted association between ≥2 valves with grade 2 regurgitation and cumulative cabergoline dose among patients treated with cabergoline only or with both cabergoline and bromocriptine (vs. only bromocriptine). [file 12902_2020_507_MOESM1_ESM.docx]

**Supplemental Tables**

**Table S1. Valvular characteristics by transthoracic echocardiography among 63 adults receiving bromocriptine only therapy for hyperprolactinemia.**

|  | **Valvular Thickening**  **N (%)** | | | | **Valvular Regurgitation**  **N (%)** | | | | |
| --- | --- | --- | --- | --- | --- | --- | --- | --- | --- |
| **Valve location** | **None** | **Mild** | **>Mild** | **Inconclusive** | **0** | **1** | **2** | **3** | **4** |
| Aortic | 56 (88.9) | 7 (11.1) | 0 | 0 | 3 (4.8) | 60 (95.2) | 0 | 0 | 0 |
| Mitral | 58 (92.1) | 5 (7.9) | 0 | 0 | 1 (1.6) | 61 (96.8) | 1 (1.6) | 0 | 0 |
| Pulmonic | 55 (87.3) | 0 | 0 | 8 (12.7) | 7 (11.1) | 53 (84.1) | 3  (4.8) | 0 | 0 |
| Tricuspid | 63 (100.00) | 0 | 0 | 0 | 4 (6.4) | 51 (80.9) | 8 (12.7) | 0 | 0 |

**Table S2. Valvular characteristics by transthoracic echocardiography among 62 adults receiving cabergoline only therapy for hyperprolactinemia.**

|  | **Valvular Thickening**  **N (%)** | | | | **Valvular Regurgitation**  **N (%)** | | | | |
| --- | --- | --- | --- | --- | --- | --- | --- | --- | --- |
| **Valve location** | **None** | **Mild** | **>Mild** | **Inconclusive** | **0** | **1** | **2** | **3** | **4** |
| Aortic | 52 (83.9) | 10 (16.1) | 0 | 0 | 7 (11.3) | 51 (82.3) | 4 (6.5) | 0 | 0 |
| Mitral | 59 (95.2) | 3 (4.8) | 0 | 0 | 1 (1.6) | 59 (95.2) | 2 (3.2) | 0 | 0 |
| Pulmonic | 55 (88.7) | 0 | 0 | 7 (11.3) | 6 (9.7) | 41 (66.1) | 15 (24.2) | 0 | 0 |
| Tricuspid | 62 (100.00) | 0 | 0 | 0 | 4 (6.5) | 47 (75.8) | 11 (17.7) | 0 | 0 |

**Table S3. Valvular characteristics by transthoracic echocardiography among 49 adults who received both bromocriptine and cabergoline therapies for hyperprolactinemia.**

|  | **Valvular Thickening**  **N (%)** | | | | **Valvular Regurgitation**  **N (%)** | | | | |
| --- | --- | --- | --- | --- | --- | --- | --- | --- | --- |
| **Valve location** | **None** | **Mild** | **>Mild** | **Inconclusive** | **0** | **1** | **2** | **3** | **4** |
| Aortic | 47 (95.9) | 2 (4.1) | 0 | 0 | 4 (8.2) | 43 (87.8) | 2 (4.1) | 0 | 0 |
| Mitral | 40 (81.6) | 9 (18.4) | 0 | 0 | 1 (2.0) | 44 (89.8) | 3 (6.1) | 1 (2.0) | 0 |
| Pulmonic | 40 (81.6) | 1 (2.0) | 0 | 8 (16.3) | 2 (4.1) | 39 (79.6) | 8 (16.3) | 0 | 0 |
| Tricuspid | 48 (98.0) | 1 (2.0) | 0 | 0 | 4 (8.2) | 38 (77.6) | 7 (14.3) | 0 | 0 |

**Table S4. Age-sex-adjusted association of ≥1 valve with grade 2 regurgitation among patients treated with cabergoline only or with both cabergoline and bromocriptine (vs. only bromocriptine).**

|  | **Odds Ratio for Mild Valvular Regurgitation**  **(vs. Bromocriptine only)** | **95% Confidence Interval** | **P value** |
| --- | --- | --- | --- |
| Cabergoline | 3.1 | 1.3 - 7.4 | 0.01 |
| Cabergoline and bromocriptine | 1.6 | 0.6 - 4.0 | 0.34 |

**Table S5. Age-sex-adjusted association of ≥1 valve with grade 2 regurgitation and cumulative cabergoline dose among patients treated with cabergoline only or with both cabergoline and bromocriptine (vs. only bromocriptine).**

|  | **Odds Ratio for Mild Valvular Regurgitation**  **(vs. Bromocriptine only)** | **95% Confidence Interval** | **P value** |
| --- | --- | --- | --- |
| Cumulative dose ≤ 115 mg | 2.7 | 1.2 - 6.4 | 0.02 |
| Cumulative dose > 115 mg | 1.9 | 0.8 - 4.7 | 0.16 |

**Table S6. Age-sex-adjusted association between ≥2 valves with grade 2 regurgitation among patients treated with cabergoline only or with both cabergoline and bromocriptine (vs. only bromocriptine).**

|  | **Odds Ratio for Mild Valvular Regurgitation**  **(vs. Bromocriptine only)** | **95% Confidence Interval** | **P value** |
| --- | --- | --- | --- |
| Cabergoline | 8.0 | 0.9 - 69.0 | 0.06 |
| Cabergoline and bromocriptine | 7.2 | 0.8 - 64.0 | 0.08 |

**Table S7. Age-sex-adjusted association between ≥ 2 valves with grade 2 regurgitation and cumulative cabergoline dose among patients treated with cabergoline only or with both cabergoline and bromocriptine (vs. only bromocriptine).**

|  | **Odds Ratio for Mild Valvular Regurgitation**  **(vs. Bromocriptine only)** | **95% Confidence Interval** | **P value** |
| --- | --- | --- | --- |
| Cumulative dose ≤ 115 mg | 7.5 | 0.9 - 64.5 | 0.07 |
| Cumulative dose > 115 mg | 7.8 | 0.9 - 68.3 | 0.06 |
